# The Value Chain of *Moringa oleifera* Plant and the Process of Producing Its Biodiesel in Ghana

**DOI:** 10.1155/2022/1827514

**Published:** 2022-07-18

**Authors:** Mohammed Takase, Paul Kwame Essandoh, Richard Kwadzo Asare, Kizzie-Hayford Nazir

**Affiliations:** ^1^Department of Environmental Science, School of Biological Sciences, University of Cape Coast, Cape Coast, Ghana; ^2^Ho Polytechnic, Ho, Ghana; ^3^Department of Biochemistry, School of Biological Sciences, University of Cape Coast, Cape Coast, Ghana

## Abstract

*Moringa oleifera* grows well in different parts of Africa, including Ghana, and throughout the world as it can tolerate moderate cold and severe drought. Dubbed as the “Miracle Tree,” Moringa has a number of nutritional, pharmaceutical, and cosmetic applications. It is also used as a cure for diabetes and various forms of cancer. Another promising use of this plant is the production of biodiesel. However, such application demands vast cultivation areas and this can lead to competition with the cultivation of crops for food, forestry, and environmental protection. Furthermore, it requires suitable cultivation schedules that favor the growth of pods containing oil-rich seeds. The present work investigates the availability of land for Moringa cultivation in Ghana to increase the energy production from the plant. The study reports on an overview of the soil, water, and climatic conditions that favor *Moringa oleifera* cultivation and oil production, the conversion of *Moringa oleifera* oil to biodiesel, and the performance of *Moringa oleifera* biodiesel versus mineral diesel as well as the commercial enhancement of the Moringa product.

## 1. Introduction

Global population growth has increased the energy consumption chain, available energy demands, and resource production. The world population and available energy production crops are exceeding their carrying capacity [1]. The immediate concerns or questions that come into mind are (1) how will the energy demand in the future be like as the human population grows exponentially? (2) how will the available crop resources sustain the energy demands of the rapid population growth, and (3) how accessible are lands for sustainable energy production to augment the rapid population growth as conflicts over land are rampant. Thus, rapid population growth demands rapid but green and sustainable energy production. On the other hand, rapid population growth leads to the rapid depletion of energy resources. An increase in population requires large-scale energy crop production, and a rapid population increase demands large land availability for green and sustainable energy production.

Nevertheless, to successfully produce sustainable and readily available energy crops for green energy production, Achten et al. [2] noted that Moringa, which has excellent, yellowish, and non-dry oil, can be an alternative to the energy production chain. Moringa can thrive in all weather and can produce large and distinctive fruits as well as firewood [3–7].

However, providing sufficient lands for *Moringa oleifera* cultivation as bioenergy crop types and proper management practices would help protect the environment and sustainable development of *Moringa oleifera* bioenergy [8–10]. According to Achten et al. [2], energy is key for development, the existence of ecosystems, good quality of life, and society. This study, therefore, examines the prospect of land use for *Moringa oleifera* cultivation in Ghana and the implication on other crops and the country's economy. The study highlights the importance of *Moringa oleifera* farming based on a green plantation and some key environmental issues in Ghana.

### 1.1. The Moringa Plant

*Moringa oleifera* grows fast with a life span of around 1–3 months, a height of 2.5 m, with its pod weighing 120 g per pod, length of the pod being 45–50 cm, and a harvest of 1000–1200 pods per tree. The seeds are used to make oils, while the roots and leaves are used to make powders. The bark is whitish-grey and is encased in thick cork. The tree has open crowns with hanging, weak limbs, and the leaves form fluffy foliage of tripinnate leaflets. The flowers are aromatic and reproduce asexually with five uneven, lightly veined, yellowish-white petals surrounding them. The blooms are 1.0–1.5 cm long and 2.0 cm (3/4 in) in width. The blooms grow in spreading or hanging flower clusters on thin, hairy stalks with a length of 10–25 cm. The flower appears once a year, in late spring and early summer, and blooms during the first 6 months of planting. The fruit is about 20–45 cm long, a three-sided brown capsule containing dark brown, spherical seeds 1 cm in diameter [11].

### 1.2. Regional Production of *Moringa oleifera*

Regionally, Moringa serves as a source of food for many people [12]. In India, for example, seeds are used to produce oil. The seedcakes are obtained from the leftovers of crushing the seeds for oil. The seeds contain 58% crude protein. According to Jingura et al. [13] and Joshi et al. [14], some districts of Kenya as well as the Salem district in South India have widely cultivated *Moringa oleifera* for commercial purposes. Studies by Quintero et al. [15] indicate that the oil can be used for biodiesel, food, and nonedible applications. Biodiesel made from *Moringa oleifera* seed oil has a stronger oxidative ability, high cloud point, and a cetane number of around 67, which is higher than most biodiesels. Biodiesel from *Moringa oleifera* oil is safe to transport and can be stored longer. Ghana has, however, focused on boosting the productivity of *Moringa oleifera* per hectare [16].  Table 1 shows the major biofeedstock production and five primary stocks as well as biofuel energy [17, 18].

### 1.3. Worldwide Cultivation of *Moringa oleifera*

**Figure 1:**
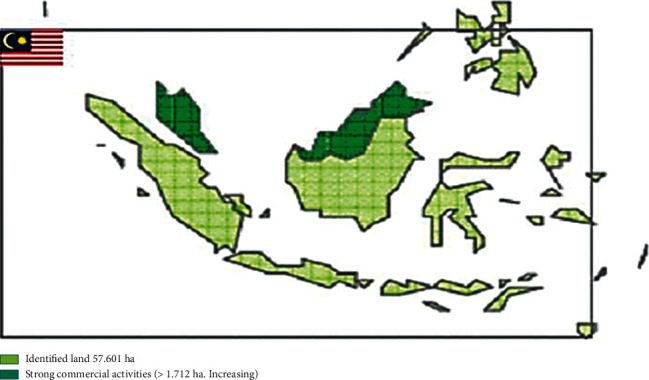
Land for *Moringa oleifera* in Ghana [20].

*Moringa oleifera* cultivation is in its fundamental stage of evolution. For example, entrepreneurs are involved in the plantation on a commercialized scale. Farmers cultivate *Moringa oleifera* by using vegetative means or seeds or combining both methods. So far, *Moringa oleifera* cultivation in Ghana has been somewhat low, and project activities are still slow. Land for *Moringa oleifera* agriculture is shown in Figure 1 [19]. The major cause for the slow pace of growth appears to be high prices of land acquisition (in comparison to other states) and scarcity of labor. According to local experts, small-scale farmers are yet to begin growing *Moringa oleifera* [20]. Currently, the entire land area covered by moringa is about 1,712 hectares. By 2025, it is estimated that the area will be increased to about 57,601 hectares [21, 22]. The Ministry of Agriculture has, however, granted 300 hectares for the *Moringa oleifera* pilot research project. Meanwhile, some foreign oil companies have announced their intentions to establish *Moringa oleifera* plantations in Ghana [23, 24]. Notwithstanding 300 hectares of land granted by the Ministry of Agriculture, some local private enterprises have already successfully planted *Moringa oleifera* on lands ranging 400 and 1000 hectares.

### 1.4. *Moringa oleifera* Cultivation in Ghana

In Ghana, Moringa is being encouraged in many localities and more than 10,000 farmers so far are cultivating *Moringa oleifera* using enhanced agronomic practices, especially in the northern parts of the country. The Department of Crop Science, School of Agriculture, University of Cape Coast has also explored the prospects of commercializing *Moringa oleifera* as well as some entrepreneurial and economic issues [20, 29, 30].

### 1.5. Land and Cultivation of *Moringa oleifera* in Ghana

Across all 278 districts in Ghana, most farmers plant Moringa on an area ranging from 0.25 to 1.5 hectares. This yields about 554 metric tons per year. Takase et al. [31] reported that 45% of Ghanian agricultural land is less productive, but Moringa has been utilized to improve the soil's physical, chemical, and biological qualities. With a plant population of 1600 per hectare, a replacement of 2.5 × 2.5 m is suitable. Using a well-drained sandy loam soil, the optimum spacing can be 1.33 million plants per hectare (i.e., 5 × 15 cm) [32]. In Ghana, various organizations are training Moringa farmers for the production of Moringa leaves and seeds. For example, the US is empowering over 1000 farmers in northern Ghana to produce Moringa for both the leaves and seeds. The Ghana Permaculture Institute likewise trained farmers to use Moringa in cropping.

According to Achten et al. [2], under high temperature and precipitation conditions in Ghana, applying fertilizers to the soil will promote plant development and chemical composition. And therefore planting Moringa at a high density will enhance biomass output. Planting at a density of 435,000 plants per hectare can result in increased biomass buildup cutting intervals. The crop may provide cattle with a sufficient amount of crude protein, and the tree is rich in other nutrients rendering it a potentially useful source of supplementary feed when there is insufficient natural forage due to drought. Moringa may provide a high yield even at a high plant density. Normally, during high precipitation and temperature periods, applying fertilizer should be considered to promote plant development and chemical composition.

### 1.6. The *Moringa oleifera* Plant and Its Cultivation Processes

*Moringa oleifera* is a fast-growing, drought-resistant plant belonging to the *Moringaceae* family which is mostly grown for its leaves and fruits [33]. *Moringa oleifera* thrives in tropics and subtropics regions with some climatic factors including annual precipitation ranging from 760 to 2500 mm and temperatures and mean annual temperature of 18 to 28°C. *Moringa oleifera* grows well in every type of soil at a pH of 4.5 to 8 and altitudes of 2000 meters. *Moringa oleifera* does have a wide range of morphological variations and diversity in several features [34]. The roots formation takes place about 20 days after planting and this enables immature plants to withstand drought. The tree can thrive in marginal lands (absence of fertilizer). A single tree can yield 15,000 to 25,000 seeds in a year with an average weight of 0.3 g per seed. Normally, early blooming cultivars yield pods in 6 months, while other kinds take more than a year to yield. Within 6 months of trimming, branches do generate new pods. For example, India's largest *Moringa oleifera*, yield about 1.1 to 1.3 million tons of delicate fruits from an area of 380 km^2^ annually [33].

**Figure 2:**
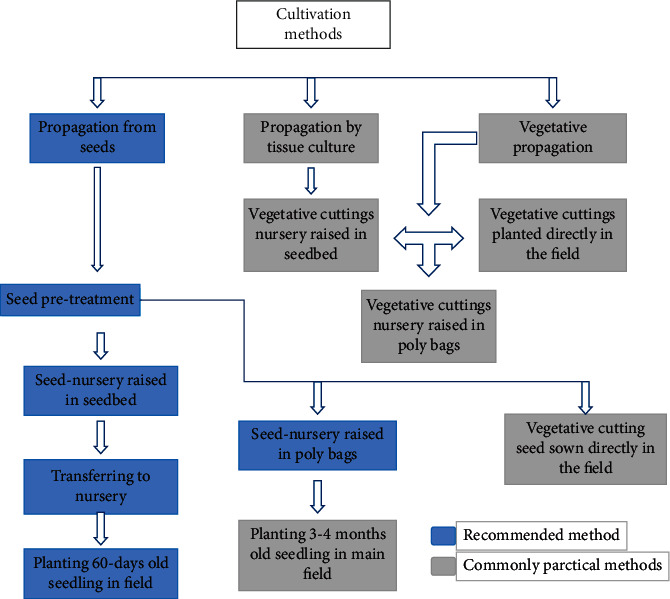
Flow chart of *Moringa oleifera* cultivation methods [34].

Cultivation processes are very important factors in obtaining favorable yields from *Moringa oleifera* [34, 35]. There is a common misperception that *Moringa oleifera* plant requires very little water and no pesticide or fertilizer input [36]. The plant requires substantially less inputs and upkeep than many other energy crops [37]. Yields can indeed be higher than the average if proper planting methods and agronomic practices are adhered to [36].  Figure 2 illustrates the suggested cultivation methods, inputs, and associated themes for *Moringa oleifera* production.

On the basis of the various estimates and data, the range of yield of *Moringa oleifera* seeds is presented in Table 2 [7, 34, 38].

### 1.7. The Biology of *Moringa oleifera* Plant

*Moringaoleifera* is a deciduous tree and big shrub or small tree that grows to a height of 3–5 m and on some occasions can grow 8–10 m. When the tree is chopped, it has smooth grey bark, which when dried releases a whitish-colored liquid latex that becomes brittle and discolored [39]. Its leaves are green with pale-green stripes of about 1 cm long and 3 cm wide with 3–7 lobes and spiral phyllotaxis.

The stomata and hypostomatic are of paracytic type. Insects, flies, thrips, ants, and bees pollinate the flowers [40]. The length of the petiole ranges from 3 to 18 mm. The inflorescence develops on the leaf axis and produces 10 or more ovoid fruits. During warmer seasons, the female flower becomes somewhat larger when there is an imbalance of a pistil late or stamina. It has more female flowers. Fruits are produced throughout the year and the range of production may be attributed to places with low and high rainfall as well as soil richness. After the seed has developed and the fleshy portion dries, a three-bivalve coccid is created. The capsule transforms from green to yellow within 2–3 months after it is riped for plucking. The inner seeds are black and weigh about 343 g per 1000 seeds.

### 1.8. Moringa Seeds

*Moringa* seeds are planted during the rainy season and are capable of germinating and growing without irrigation, but only for commercial purposes. They are three-angled with an average weight of about 0.3 g. The seeds are also three-winged with wings developing from the base of the seeds to the tip (2–2.5 cm in length and 0.4–0.7 cm in width). The kernel accounts for 70%–75% of the weight of the seed. To extract oil from the seeds, seeds moisture is maintained and the seeds are stored and dried at room temperature to avoid seasonal variation in the temperature [38].

### 1.9. *Moringa oleifera* Oil

*Moringa oleifera* oil has high monounsaturated to saturated fatty acid, tocopherols, and proteins as well as sulfated amino acids [37]. Specifically, the oil contains 40% of high-quality fatty acid (oleic acid >70%) and Behen oil or Ben oil. Ben oil can be recovered from the seeds of moringa by solvent extraction and CO_2_ supercritical extraction. In diets, the oil can be used in place of olive oil [25].  Table 3 shows *Moringa oleifera* oil components and Table 4 shows *Moringa oleifera* oil yield (kg oil/ha) [6, 25–28, 38, 41].

### 1.10. Soil Water and Climate Conditions

*Moringa oleifera* grows in a variety of soil. However, the plant grows very well in neutral to slightly acidic soil (pH. 6.3–7.0) thus, sandy or loamy soil drains properly. The minimum annual rainfall needed is 250 mm, with a maximum of about 3,000 mm, although in wet soil, the roots decay.

Obviously, *Moringa oleifera* would require more water and hence, will require the establishment of irrigation facilities for all-year-round cultivation [41]. *Moringa oleifera* has the potential to fertilize the soil in many ways and environments [21, 42]. However, over the last four decades, soil erosion has been a severe environmental challenge to the long-term viability and productivity of agriculture [42]. Over the period, some of the world's productive land has been lost due to erosion and this loss is continuing at a rate of more than 10 million hectares per year [43].

And because of the increased interest in *Moringa oleifera*, most initiatives are no longer designed just for soil erosion prevention, but also for economic and social advantages [44–46]. Most operations are marked by new agronomical and technological issues due to emerging manufacturing and conversion methods as well as adopting new rural business practices and the emergence of environmental problems concerning long-term sustainability [47, 48].

### 1.11. Extraction of *Moringa oleifera* Oil

The predominant component of the seed of *Moringa oleifera* is the oil which contributes 36.7% of its weight. The oil can be extracted using various methods. Some of these include solvent extraction using *n*-hexane. However, cold press extraction is also possible, which may result in a yield of about 69% of the oil samples from the seeds. Normally, the oil meant for consumption is extracted by heating de-husked seeds in water and scooping the oil from the water's surface.

The quality of *Moringa oleifera* oil is very high when compared with other vegetable edible oils. It tastes nutty and is similar to olive oil. The oil is more reliable and even nutritionally healthier than most other frying oils. It is a rich source of dietary energy. Studies have shown that even a small amount of oil added to children's diets has been found to give a more diversified and healthy diet since the oil is high in vitamins A, C, and E [24, 30, 48].

**Figure 3:**
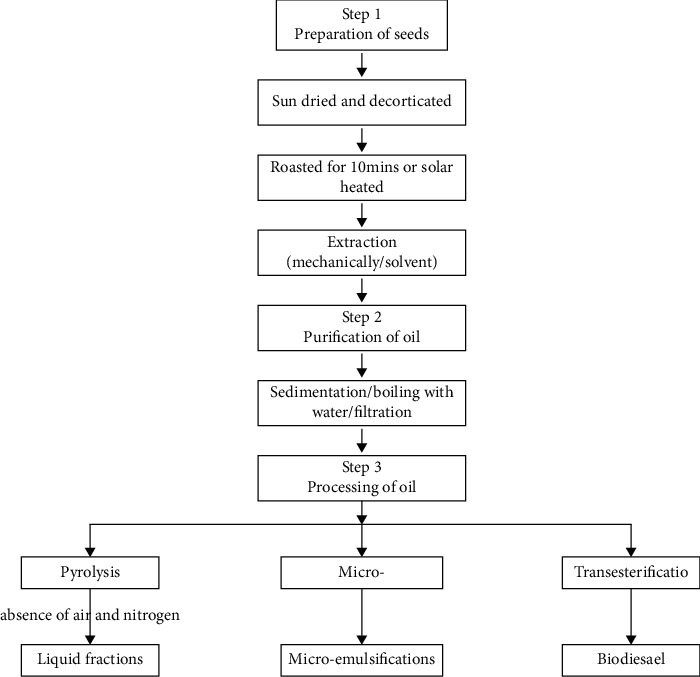
Process flow for a typical oil refining plant [7].

Figure 3 shows a typical process for refining *Moringa oleifera*. The general manufacturing process for *Moringa oleifera* oil is as follows: Sowing⟶Cultivation⟶Harvest⟶ Seed Dehulling and Cleaning⟶Oil Extraction⟶Oil Filtration and Purification⟶ Oil Refining.  Table 5 shows the percentage of oil extracted from *Moringa oleifera* seeds.

### 1.12. Conversion of *Moringa oleifera* Oil into Biodiesel

Moringa biodiesel can be produced using various methods and conditions below.

### 1.13. Methods of Producing Moringa Biodiesel

A number of methods are available for producing biodiesel from *Moringa oleifera* oil [49, 50]. Crude oils are modified in order to reduce their viscosities for the product to be compatible with diesel engines. Among the available methods, blending, microemulsions, thermal cracking, and transesterification are commonly used [50].

### 1.14. Blending

Blending is the process of reducing the concentration of solute in a solution usually by mixing it with more solvent. Crude oils can be mixed directly or diluted with diesel in order to improve viscosity. The resulting blended solution is mixed well to ensure that all parts of the solution are identical. A blend of 20% to 40% of vegetable oil with diesel has been tested and proven promising [50]. Studies on successful blending using various nonedible resources such as rubber seed oil, jatropha, *Putranjiva roxburghii*, and *Moringa oleifera* seed oil with diesel for diesel engines have been reported [50].

### 1.15. Micro-Emulsification

Microemulsion is another approach to modifying *Moringa oleifera* oils to fuel. Microemulsions are clear, stable isotropic fluids with three components (oil phase, aqueous phase, and surfactant). The aqueous phase contains salts or other ingredients while the oil phase contains a complex mixture of different hydrocarbons. This ternary phase improves the spray characteristics through explosive vaporization of low boiling constituents in the micelles. Microemulsions using butanol, hexanol, and octanol have been reported to meet the maximum viscosity limit for diesel engines. Microemulsions can be prepared with or without diesel. The process is considered a reliable approach for reducing the viscosity of vegetable oils [51, 52].

### 1.16. Pyrolysis

Pyrolysis, another method of modifying crude *Moringa oleifera* oil, is the process by which one substance is converted to another by means of heat or catalyst in the absence of air or oxygen. The pyrolyzed materials have low viscosity. However, the pour point, flash point, and cetane number of pyrolyzed materials are greater than those of conventional diesel. The materials have equivalent calorific value as diesel with an acceptable amount of sulfur, copper corrosion, and water, but unacceptable carbon residue and ash content [49]. Depending on the operating conditions, the process of pyrolysis can be divided into three namely conventional, fast, and flash pyrolysis [50]. Much studies on pyrolysis for biodiesel production from vegetable oils have been conducted and proven successful [53].

### 1.17. Transesterification

Transesterification is the chemical process involving triglycerides and alcohol in the presence of a catalyst to form esters and glycerol as the backbone [52]. Transesterification process involves three consecutive reversible reactions namely, triglycerides are first converted to diglycerides, and the diglycerides are then converted to monoglycerides and glycerol backbone. Catalyst is usually used to enhance the conversion [54, 55]. Different catalysts are used for transesterification reactions. Examples include magnesium, calcium oxides, carbonates of basic and acidic, macro-reticular organic resins, alkane alumina, sulfuric acids, sulfonic acid, and dehydrating agents as co-catalysts. These catalysts are generally grouped into alkalis, acids, or enzymes [50]. The alkali catalysts are usually preferred over the acids and enzymes due to the higher reactivity and milder process conditions [56].

### 1.18. Forms of Transesterification

Depending on the type of catalyst, transesterification is grouped into either acidic or alkaline [50]. Acid-catalyzed transesterification is also known as esterification. The choice of catalyst is normally informed by the amount of free fatty acids and the water content of the oil. Usually, low free fatty acids with low water content oils require alkaline transesterification, while waste and nonedible oils with high free fatty acids require acid or esterification. Below are the highlights of the various transesterification processes.

#### 1.18.1. Alkali-Catalyzed Transesterification

**Figure 4:**
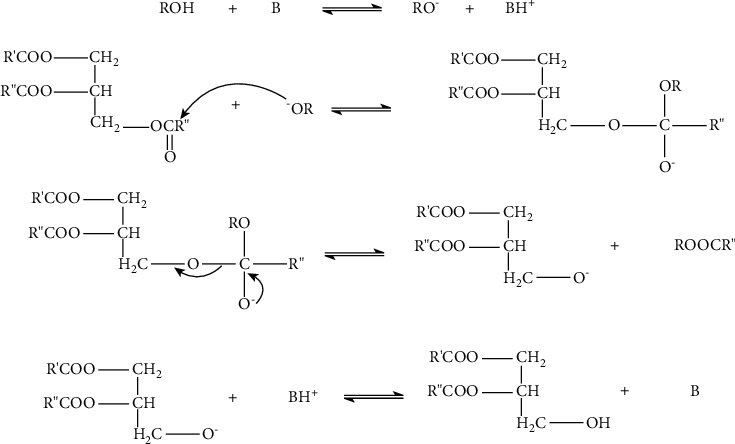
The reaction mechanism of base catalyst [51].

Alkali-catalyzed transesterification is one of the commonest processes. This is because the process proceeds faster than the other catalysts (acids and enzymes). The reaction mechanism of alkaline transesterification proceeds in three main steps (Figure 4) [51] as follows: first, there occurs a reaction of a carbonyl carbon atom with anion from alcohol to form a tetrahedral intermediate from which alkyl esters and corresponding anions of diglycerides are formed. A different catalytic cycle is started when the catalyst reacts with a second molecule of alcohol. The diglycerides and monoglycerides are then converted to alkyl esters and glycerol [52]. Takase [53] studied the reaction mechanism of transesterification of *Jatropha curcas* oil as a nonedible and has proven the three-step reaction mechanism. Base-catalyzed transesterification is most suitable for low FFA and water content oils (less than 1% w/w) [31].

#### 1.18.2. Acid-Catalyzed Transesterification

**Figure 5:**
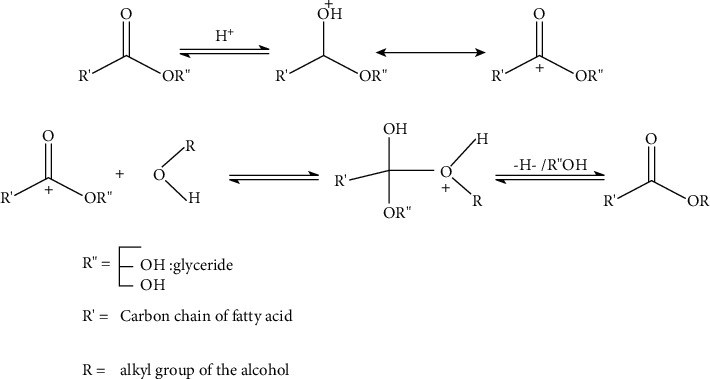
The mechanism of acid-catalyzed transesterification [51].

Acid-catalyzed transesterification is not as popular as base-catalyzed. This is due to the slow rate of reaction and the high methanol to oil ratio required. Acid catalysts are also characterized by low activities with high temperatures during transesterification [53]. The long reaction time makes the process impractical and uneconomical [51]. The relative advantage of using acid catalysts for transesterification is the tolerance toward the presence of high FFAs in the feedstock. For instance, acid catalysts can directly produce biodiesel from low-cost lipid feedstock with FFA greater than 6% [56, 57]. Liquid acid catalysts (such as sulfuric acid) have tolerance and are less sensitive to high FFA oils and can simultaneously conduct esterification and transesterification by giving relatively high yields of esters [57]. The mechanism of acid-catalyst transesterification is shown in Figure 5 [51]. The process involves protonation of the carbonyl group of esters which promotes the formation of carbon cations after the nucleophilic attack of alcohol which produces a tetrahedral intermediate. The intermediate eliminates glycerol to form a new ester and regenerates the catalyst. Acid-catalyzed transesterification can be carried out in the absence of water.

A report by Takase et al. [51] using solid super acidic catalysts of sulfated tin and zirconium oxides and tung zirconia for transesterification of soybean oil with methanol at 475 to 575 K indicated the suitable conditions for acid catalyst.

#### 1.18.3. Enzyme-Catalyzed Transesterification

Enzymatic transesterification using lipase catalyst is becoming more attractive as a result of the easy product separation, less wastewater, easy glycerol recovery, and absence of side reactions. Lipases are generally known to act better on long-chain fatty acids than short-chains. Biocatalysts are more expensive and their regeneration and reusability are limited by longer operation time [58]. The reaction yields of enzyme-catalyzed transesterification are unfavorable and thus, render the enzyme process uneconomical [53].

Studies on lipase-catalyzed transesterification using Novozym (*Candida* Antarctica immobilized on acrylic resin) for biodiesel have indicated the process as promising [53]. However, the complexity of the lipase purification process makes the process uneconomical and hinders its larger scale application.

#### 1.18.4. Non-Catalytic Supercritical Alcohol

Non-catalytic supercritical alcohol is a new method of producing biodiesel. Under this supercritical condition, the reaction process could be completed in a few minutes with relatively high yields. Studies indicate that increasing the ratio of reaction temperature to a supercritical temperature can have a favorable influence on ester conversion [53]. The main advantage of this method is the fact that purification of biodiesel is much easier as no catalyst is required during the supercritical reaction processes. Soap formation or saponification reaction is also minimal. The presence of water which has a negative impact when using conventional transesterification is also no longer an issue [59]. However, the main problem of this process is the need for high temperature and pressure which consequently increase the cost of production. Cosolvents such as CO_2_, hexane, propane, calcium oxide, and subcritical alcohols can, however, be added to reduce the operating temperature and pressure during reaction [52].

Takase et al. [53] indicate that 100% yield of biodiesel can be obtained in 4 min using supercritical methanol at a temperature of 320°C, the pressure of 8.4 MPa, and 43:1 molar ratio of methanol to oil.

### 1.19. Factors Affecting *Moringa oleifera* Biodiesel Production

#### 1.19.1. Reaction Temperature

The rate of reaction is strongly affected by the reaction temperature. A high reaction temperature can decrease the viscosity of oil which leads to an increase in reaction rate. Ideally, the reaction temperature should be less than the boiling point of the alcohol in order to ensure that the alcohol evaporates. If the reaction temperature exceeds the optimum limit, the yield of biodiesel decreases since higher reaction temperatures accelerate saponification reaction with a consequent reduction in yield [50]. Depending on the alcohol and the oil, the maximum yield is mostly obtained at temperatures between 60 and 80°C [57].

### 1.20. Molar Ratio of Alcohol to Oil

The stoichiometric ratio of transesterification reaction requires 3 mols of alcohol to 1 mol of triglyceride to yield 3 mols of fatty acid esters and 1 mol of glycerol. Excess alcohol is to ensure that the oil is completely converted to esters under favorable conditions. High alcohol to oil ratio can result in a greater ester conversion in a relatively shorter period. The alcohol to oil molar ratio is associated with the type of catalyst used. For a base-catalyzed system where the free fatty acids are usually less than 1%, a molar ratio of 5:1 or 6:1 is sufficient to convert the triglyceride to biodiesel [53]. However, when the amount of the free fatty acids in the oil is high (>1%), a molar ratio of 20:1 or more is usually needed (acid-catalyzed transesterification) [53].

### 1.21. Catalyst Concentration

The concentration of catalyst is a very important factor that influences the conversion of fatty acids to esters. Base catalysts are usually preferred to acid catalysts because of the high reactivity with low process temperatures [50]. Ideally, an increase in the catalyst concentration leads to increases in the conversion of triglycerides for maximum yield. However, beyond the maximum yield, the excess catalyst leads to yield reduction as a result of soap formation. There are varieties of base catalysts with high catalytic performance. For instance, Takase et al. [53] indicated that sodium methoxide is one of the effective base catalysts for transesterification. Studies indicate that concentration of NaOH in the range of 1.0% to 1.4% (w/w) can produce 90% to 98% methyl esters [50] while KOH concentration between 0.55% and 2.0% (w/w) could result between 95% and 99% biodiesel yield [49].

### 1.22. Reaction Time

The conversion of triglyceride to esters increases with reaction time. Usually, the reaction begins slowly at the initial time as a result of mixing and dispersion of alcohol onto the oil. The reaction, however, proceeds faster with time until the maximum yield is reached [51, 60, 61]. For base catalysts, the yield of esters reaches maximum ideally in 2 h or less [102]. Acid catalysts, however, require a longer reaction time (>2 h) [49]. Depending on the catalyst (acidic), the reaction time required for the conversion of triglycerides to biodiesel could range from 4 to 24 h [51]. An excess reaction time can lead to a reduction in the product yield due to backward reaction and cause the excess fatty acids to form soaps [50].  Table 6 shows catalyzed transesterification of vegetable oils with optimized reaction variables.[53]

### 1.23. Biodiesel Characterization from *Moringa oleifera* Oil

*Moringa oleifera* biodiesel properties have been studied and are seen to fall within ASTM D6751 specification.  Table 7 shows the properties of *Moringa oleifera* biodiesel compared with conventional diesel and ASTM D6751 standard. From Table 7, the properties of *Moringa oleifera* biodiesel were found to be within the international standards for direct usage as a fuel for diesel engines [37, 44]. By specific, the acid value of biodiesel is largely a measure of free fatty acid. If the acid value is more than 0.8 mg KOH per g, free fatty acid may be deposited on the fuel system, resulting in a reduced lifetime for the fuel pumps and filters [45]. High viscous fuels might result in inefficient fuel combustion resulting in deposit development. As shown in Table 7, virtually, the parameters of *Moringa oleifera* biodiesel including acid value, kinematic viscosity, flash point, specific gravity, water content, and ash content are within those of conventional diesel and meet the ASTM D6751 standard [7, 41] (Table 7).

### 1.24. Performance Comparison of Mineral Diesel and *Moringa oleifera* Biodiesel

**Figure 6:**
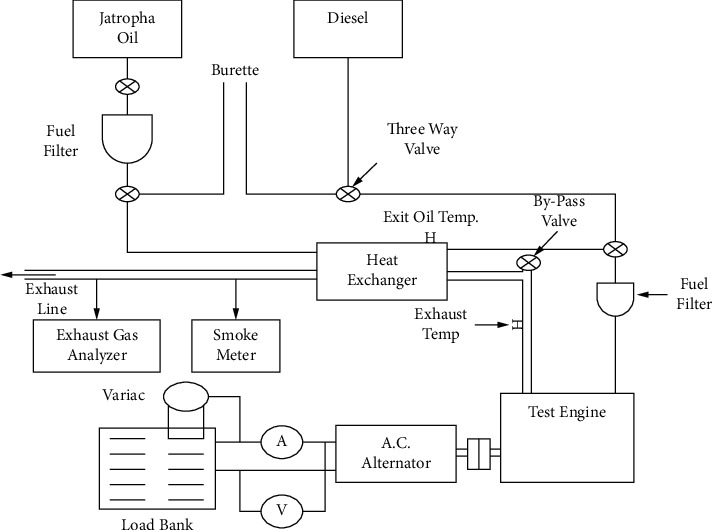
Schematic diagram and the experiment setup [24].

Studies have been carried out on the performance of *Moringa oleifera* biodiesel compared with mineral diesel in the engine [32]. The study had a schematic representation of the engine examined. Before the study, the oil sump was filled with new lubricating oil. A single-phase 220 V AC alternator was connected to the engine. The alternator was used to supply power to the engine via a resistive load bank. The load bank comprised eight heating coils (1000 W each). A variac was linked to one of the heating coils so that the load could be accurately regulated by altering the voltage in one of the coils of a load bank.  Figure 6 shows the typical schematic architecture of the experimental setup of performance and emission of *Moringa oleifera* biodiesel. The experimental setup consisted primarily of two fuel tanks (diesel and *Moringa oleifera* oil), an exhaust gas line, performance and emissions measuring equipment, a fuel conditioning system and heat exchanger, and a by-pass line. Two gasoline filters were installed adjacent to the fuel tank so that if one filter becomes clogged, the fuel supply could be cleaned without halting the engine [36]. The engine started with diesel and then converted to *Moringa oleifera* biodiesel after it has warmed. A by-pass valve was installed in the exhaust gas line before the heat exchanger to maintain the temperature of the *Moringa oleifera* oil within a range of 80–90°C. Following that, the diesel and *Moringa oleifera* biodiesel's performance on the engine was evaluated for a variety of physical, chemical, and thermal characteristics (Table 8).

*Moringa oleifera* biodiesel had a higher density, cloud point, and pour point than diesel. In cold climatic conditions, the cloud point and pour point values indicated that *Moringa oleifera* biodiesel could be unsuitable as diesel fuel. When compared with mineral diesel, the flash and fire points of *Moringa oleifera* oil were relatively high. As a result, *Moringa oleifera* biodiesel is safe to use. Increased carbon residue from *Moringa oleifera* biodiesel could result in increased carbon deposits in the engine's combustion chamber. Diesel and *Moringa oleifera* oil were tested for CHNOS (carbon–hydrogen–nitrogen–oxygen–sulfur). And as *Moringa oleifera* biodiesel had a low sulfur concentration, it produced less SOx.

The presence of oxygen in fuel increases combustion characteristics and emissions while decreasing the fuel's calorific value. When compared with mineral diesel, *Moringa oleifera* biodiesel had a calorific value of about 90%. The NO_x_ emissions were also influenced by the fuel's nitrogen concentration (formation of fuel NO_x_).

## 2. Conclusion

Energy is said to be a key factor in Ghana's development and provides vital services that improve the quality of life of its people. Meanwhile, the resources for energy production and use have resulted in major concerns on the environment (from the use of resources and pollution point of view) [51, 62]. The pollution, cost, and environmental concerns of fossil energy have been a challenge for the sustainable development of Ghana. Meanwhile, the aim of country is to foster development and prosperity through gains in energy efficiency production rather than increase consumption through a transition toward environmentally friendly use of renewable resources. This will definitely see biodiesel as the way forward. With the energy potential of nonedible resources, especially *Moringa oleifera* in Ghana, it is evident that the cultivation of *Moringa oleifera* and its subsequent use for biodiesel as a nonedible resource will help increase energy and food security. Moringa and its value change can contribute to sustainable development goals in Ghana since the plants can be grown on wastelands to reclaim them, may not compete with food crops for limited lands, are relatively cheap, available, and offers similar or even higher fuel yields, and properties as mineral diesel. Also, *Moringa oleifera* development can be used to curb the high unemployment rate through job creation, increased income of the Ghanian populace, diversifying, and enhancing the quality of the environment (pollution reduction). Replacement of fossil fuels with biodiesel from *Moringa oleifera* and other nonedible resources production will also offer the youth and children the opportunity to engage in productive businesses and enhance their standard of living. It can therefore be deduced from the study that *Moringa oleifera* is a multipurpose nonedible resource in Ghana and its commercialization can result in Ghana's economic improvement.

## Figures and Tables

**Table 1:** Production of five major feedstocks and biofuel energy yields in 2005 [6, 10].

Country and top 2 crop producers	Maize	China	Sugarcane	India	Cassava	Brazil	Soybean	Brazil	Oil palm	Indonesia
	USA		Brazil		Nigeria		USA		Malaysia	
Total production million tons	280	133	420	323	42	23	83	50	76	64
% World production	40	19	33	18	20	12	39	24	44	37
Average crop yield (3–5 tons/ha)	9.4	50	73.9	60.7	10.8	13.6	2.7	2.4	20.6	17.58
Conversion yield litres/tons (a)	399	399	74.5	74.5	137	137	205	205	230	230
Biofuel yield in gigajoules/ha (b)	3751	1995	4522	4522	1480	1863	552	491	4736	4092
Energy yield in gigajoules/ha (c)	79.1	41.1	95.4	95.4	31.2	393	18	16.1	156	135
2005 petroleum (d) replacement, % of petroleum use	2	2.4	1.8	1.8	0	0	0.1	0	0	0

**Table 2:** Range of *Moringa oleifera* yield per hectare [7, 35, 39].

Year	Range of reported yields per hectare (tons)	Most likely average yield per hectare (tons)
1	0.250–1.25	0.5
2	1–2.5	1.5
3	2.5–5.0	3
4	5–6.25	5
5	6.25–7.5	6.5

**Table 3:** Ghanaian *Moringa oleifera* oil components [6, 25–28].

Components	Weight (%)
Protein	18
Moisture	60.2
Ash	5.3
Carbohydrate	17
Fat	38
Fibre	15.5
Palmitic acid	14–15
Linoleic acid	31–43
Triglycerides of oleic acid	34–45

**Table 4:** Estimated *Moringa oleifera* oil yield (kg oil/ha) (2008–2020) [6, 39, 43].

Year	New plantations		Existing plantations	
	High yield potential	Standard yield potential	High yield potential	Standard yield potential
2008	500	200	2400	1500
2009	1500	700	2410	1505
2010	2000	1300	2420	1510
2011	2300	1500	2430	1515
2012	2400	1520	2440	1520
2013	2500	1540	2450	1530
2014	2520	1550	2460	1525
2015	25,540	1560	2470	1535
2016	2560	1570	2480	1540
2017	2580	1580	2490	1545
2018	2600	1590	2500	1550
2019	2620	1600	2510	1555
2020	2640	1610	2520	1560

**Table 5:** Percentage of oil extracted from *Moringa oleifera* seeds.

Fatty acids	Chemical formula	Mole fraction (%)
Myristic acid (C14:0)	C_14_H_28_O_2_	0.5
Behenic acid (C22:0)	C_22_H_44_O_2_	4.1
Oleic acid (C18)	C_18_H_34_O_2_	67.3
Stearic acid (C:18)	C_18_H_36_O_2_	4.5
Linolenic acid (C18:3)	C_18_H_30_O_2_	1.1
Erucic acid (C22:1)	C_22_H_42_O_2_	1.7
Arachidic acid (C20)	C_20_H_40_O_2_	5.5
Palmitoleic acid (C16:1)	C_16_H_30_O_2_	2.5
Palmitic acid (C16)	C_16_H_32_O_2_	7.9

**Table 6:** Catalyzed transesterification of vegetable oils with optimized reaction variables [56].

Alcohol type	Molar ratio of alcohol to oil	Catalyst used	Catalyst amount (%)	Optimum reaction condition	Biodiesel yield (%)
Methanol	5.9:1	H_2_SO_4_	15	60°C, 24 h	99.8
Methanol	1:1	*Rhizopus oryzae*	4	30°C, 60 h	80
Ethanol	4:1	*Pseudomonas cepacia*	10	50°C, 8 h	98
Methanol	43:1	—	—	320°C, 8.4 MPa, 4 h	100
Dimethyl carbonate	14:1	—	—	300°C, 9 MPa, 15 h	97
Methanol	3:1	—	—	290°C, 11 MPa, 15 h	99
Methanol	5:1	NaOH	1	60°C, 90 min	98
Methanol	4.2:1	NaOH	1.4	65°C, 120 min	90
Methanol	6:1	KOH	1	65°C, 60 min	99
Methanol	9:1	KOH	2	60°C, 120 min	95
Methanol	11:1	KOH	1.1	66°C, 120 min	93
Methanol	12:1	Alumina loaded KNO_3_	6	70°C, 360 min	84

**Table 7:** *Moringa oleifera* biodiesel fuel properties with ASTM D6751 standard [7].

Property and unit	Extracted *Moringa oleifera* oil	*Moringa oleifera* biodiesel	Diesel	Biodiesel standards ASTM D 6751
Acid value (mg KOH/g)	10.37	0.29	−2.6	<0.80
Flash point (°C)	60	140	68	>130
Specific gravity (g/ml)	0.92	0.89	0.85	0.86–0.90
Kinematic viscosity at 40°C (mm^2^/s)	48.2	2.9	2.6	1.9–6.0
Ash content (%)	0.09	0.01	0.01	<0.02
Water content (%)	0.05	0.01	0.02	<0.03

**Table 8:** Evaluated properties of mineral diesel and *Moringa oleifera* biodiesel as performed on the diesel engine [27].

Property	Fuel	
	Mineral diesel	*Moringa oleifera* biodiese
Density (kg/m^3^)	840 ± 1.732	917 ± 1
API gravity	36.95 ± 0.346	22.81 ± 0.165
Pour point (°C)	−6 ± 1	4 ± 1
Kinematic viscosity at 40°C (cSt)	2.44 ± 0.27	35.98 ± 1.3
Calorific value (MJ/kg)	45.343	39.071
Flash point (°C)	71 ± 3	229 ± 4
Conrad son carbon residue (%, w/w)	0.1 ± 0.0	0.8 ± 0.1
Ash content (%, w/w)	0.01 ± 0.0	0.03 ± 0.0
Carbon (%, w/w)	80.33	76.11
Hydrogen (%, w/w)	12.36	10.52
Cloud point (°C)	3 ± 1	9 ± 1
Fire point (°C)	103 ± 3	274 ± 3
Nitrogen (%, w/w)	1.76	0
Oxygen (%, w/w)	1.19	11.06
Sulfur (%, w/w)	0.25	0

## Data Availability

Data associated with the manuscript are included in the manuscript.
